# The PROMOTE study (High-protein and resistance-training combination in overweight and obesity) for short-term weight loss and long-term weight maintenance for Chinese people: a protocol for a pilot randomized controlled trial

**DOI:** 10.1186/s13063-019-3954-7

**Published:** 2020-01-08

**Authors:** Shaoyong Xu, Juan Zhang, Yuxiang Dong, Ruikun Chen, Wenlei Xu, Zhijun Tan, Ling Gao, Lei Shang

**Affiliations:** 1 Department of Health Statistics, Shaanxi Key Laboratory of Free Radical Biology and Medicine and the Ministry of Education Key Lab of Hazard Assessment and Control in Special Operational Environment, School of Public Health, Air Force Medical University of PLA, Changle West Road No. 169, Xi’an, 710032 Shaanxi China; 2 Department of Endocrinology, Xiangyang Central Hospital, Affiliated Hospital of Hubei University of Arts and Science, No. 136, Jingzhou Street, Xiangyang, 441021 Hubei China; 3 Department of Endocrinology, 3201 Hospital of Xi’an Jiao tong University Health Science Center, 783 Tianhan Road, Hanzhong, 723000 Shaanxi China; 4 Department of Clinical Nutrition, Xiangyang Central Hospital, Affiliated Hospital of Hubei University of Arts and Science, No. 136, Jingzhou Street, Xiangyang City, 441021 Hubei China

**Keywords:** Obesity, Weight loss, Weight maintenance, Chinese population, Randomized controlled trial

## Abstract

**Background:**

It is very important for clinicians and dieticians to explore reasonable weight management strategies for obese people that address both short-term weight loss and subsequent weight maintenance. We hypothesized that resistance training combined with a high-protein diet would result in similar short-term weight loss but better long-term weight maintenance than either a conventional low-fat diet control or a high-protein diet alone.

**Methods/design:**

This is an 8-week randomized parallel controlled trial followed by a 24-week observational follow-up study. A 48-week supplementary follow-up study will be carried out if necessary. The study will be conducted between June 2019 and October 2020. The 90 overweight or obese participants will be randomly assigned to the conventional low-fat diet group, the high-protein diet group and the high-protein diet and resistance training combination group. Primary outcomes are body weight change at week 8 and week 24 compared with the baseline level.

**Discussion:**

High-quality research on the effect of a high-protein diet combined resistance training on weight loss and weight maintenance is limited in the Chinese population. Our study will provide a basis for obesity management in China and will promote the development of exercise- and diet-related studies.

**Trial registration:**

Chinese Clinical Trial Registry, ChiCTR1900023841. Registered on 14 June 2019.

## Background

Obesity and overweight currently affect about a third of the world’s population [1]. In total, 38% of adults are expected to be overweight and 20% to be obese by 2030 [2]. Obesity is linked to all metabolic-related diseases and is also associated with more than 200 different complications. For example, more than 80% of cases of diabetes can be attributed to obesity, and obesity is also associated with diabetes-related deaths. Overweight and obesity also increase tumor risk, and 40% of tumors in the United States were estimated to be caused by overweight and obesity in 2014 [3]. Obesity significantly increases the risk of death. The relationship between obesity and mortality has been evaluated in several large-scale epidemiological studies. For example, a meta-analysis of more than 30 million people in 230 cohort studies showed a significant increase in all-cause mortality rate with overweight and obesity [4, 5]. The treatment of obesity and obesity-related diseases also carries a huge economic burden. In addition to direct economic expenses, there are other costs such as loss of labor and decline in household income. It is therefore important and urgent for overweight and obese people to lose weight [6]. Weight loss can significantly decrease the incidence of obesity- and overweight-related diseases and the associated mortality, reduce the incidence of cardiovascular diseases [7], significantly delay the progress of impaired glucose tolerance to diabetes mellitus [8], and improve urinary incontinence, sleep apnea, depression, quality of life and physical function.

Obesity is a complex polygenetic condition that is affected by genetic, behavioral, socioeconomic and environmental factors, but it is preventable and treatable [9]. Medication and surgery are only recommended for specific groups, and the first-line treatment for obesity is diet management combined with exercise [10]. Many studies have shown that the right diet and exercise guidance can help obese and overweight people to achieve short-term weight loss. However, the recommended dietary guidelines for weight loss vary significantly and have been revised several times in different social groups, reflecting uncertainties in the field of obesity nutrition management and the difficulty of establishing a uniform recommendation for all social groups. Unfortunately, most people who successfully achieve short-term weight loss (about 70%) tend to regain at least 50% of the weight over the next 2 years and return to their previous weight levels within 3 to 5 years [11]. It is therefore very important for clinicians and dieticians to explore reasonable weight management strategies for obese people that address both short-term weight loss and subsequent weight maintenance [12–15].

A high-protein diet can provide sufficient satiety and food taste to resist the hunger caused by calorie restriction and can therefore increase compliance. Compared with a high-carbohydrate diet, a high-protein diet has a more significant thermic effect, which increases energy consumption [16]. A high-protein diet combined with resistance training can reduce the loss of fat-free body mass during weight loss and maintain a relatively high basal metabolic rate. It can also develop good dietary management habits and improve self-efficacy in the initial weight loss stage. This improvement in self-efficacy comes from enthusiasm for diet intake management and exercise. People with low self-efficacy tend to be more likely to give up and regain weight [17]. There have been a few international studies on short-term weight loss and long-term weight maintenance linked to a high-protein diet [18], but high-quality research on the effect of a high-protein diet on weight loss in the Chinese population is very limited [19]. More importantly, there are only a few international and Chinese studies on the role of resistance training in medium- and long-term weight maintenance, and the results are inconsistent [20–23]. We hypothesized that resistance training combined with a high-protein diet would result in similar short-term weight loss but better long-term weight maintenance than either a conventional low-fat diet control or a high-protein diet alone.

The objectives of this study were therefore: 1) to verify that a high-protein diet has a similar short-term weight loss effect as a traditional low-fat diet; 2) to verify that a high-protein diet combined with resistance training can achieve better medium- and long-term weight maintenance; and 3) to explore whether the improvement in self-efficacy and the ability for diet management during weight loss is a predictive factor for medium- and long-term weight maintenance.

## Methods/design

### Study design

**Fig. 1:**
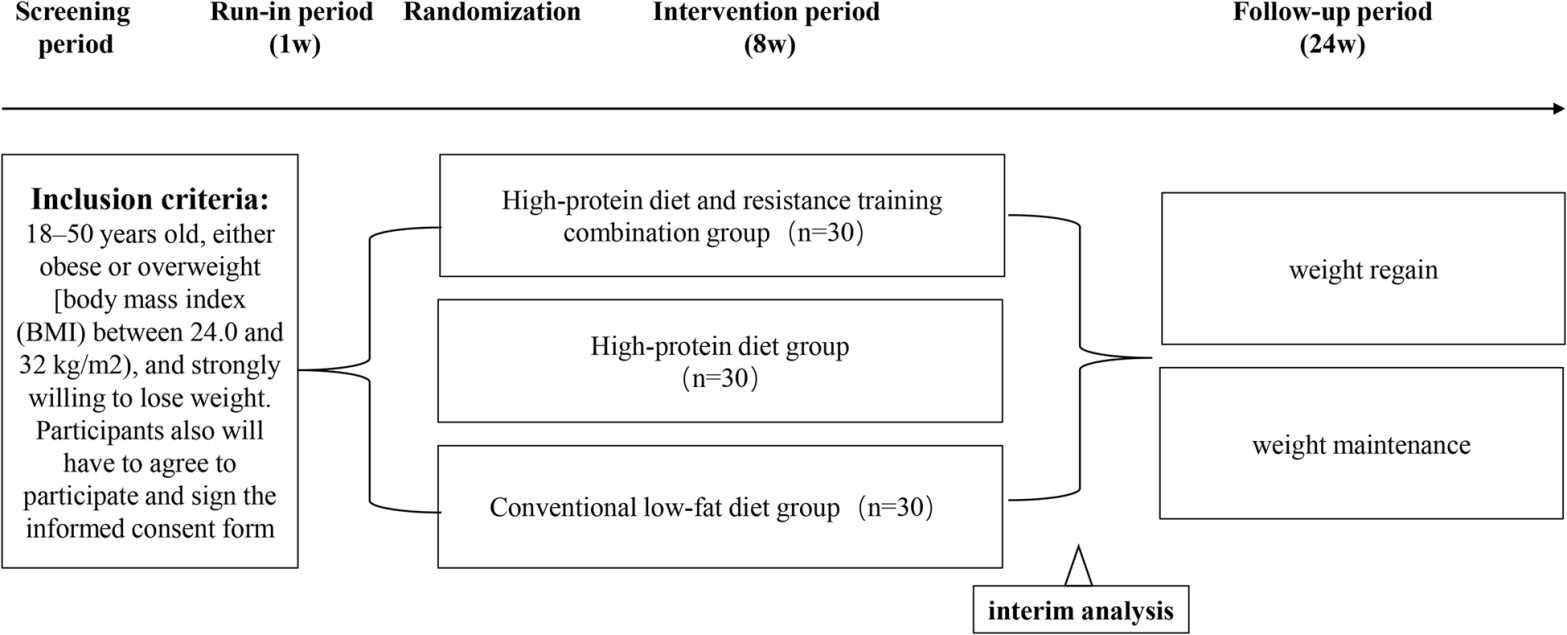
Study flowchart

The PROMOTE study (High-protein and resistance-training combination in overweight and obesity) is an 8-week randomized parallel controlled trial followed by a 24-week observational follow-up study. A 48-week supplementary follow-up study will be carried out if necessary. The enrolled participants will be randomly assigned to the conventional low-fat diet group, the high-protein diet group, or the high-protein and resistance-training combination group, and changes in body weight, body fat and metabolic indexes will be compared between the three groups in the short term (8 weeks), the medium term (24 weeks) and the long term (48 weeks). The efficacy of the high-protein and conventional low-fat diets on weight loss, fat loss and improvement of various metabolic indexes will be evaluated and compared with the efficacy of the resistance training and high-protein diet combination. After 8 weeks of intervention, the participants will be strongly recommended to continue their diet and exercise plans until the achievement of individualized weight loss goals. During the following 24 or 48 weeks of observation, the participants’ diet management knowledge will be tested, their self-efficacy evaluated, and the proportion who continued to adhere to the plans will be observed. Finally, the relationship between diet management knowledge, self-efficacy and weight loss will be explored (Fig. 1).

### Study population

The inclusion criteria will be participants who are 18–50 years old, either obese or overweight (body mass index (BMI) between 24.0 and 32 kg/m^2^), and strongly willing to lose weight. Participants also will have to agree to participate and sign the informed consent form.

The exclusion criteria will be: 1) pregnant women; 2) anyone with severe cardiopulmonary disease that cannot tolerate resistance training (cardiac function of grade III/IV); 3) anyone with diabetes, uncontrolled hypertension, recent acute infectious diseases or cancer; 4) anyone who drank excessively or had drunk heavily more than twice a week in the last month; 5) anyone with kidney disease or renal insufficiency (estimated glomerular filtration rate <90 mL/min/1.73 m^2^); 6) anyone who had stopped smoking in the past 6 months or had a recent plan to do so; 7) anyone with hypothyroidism, Cushing’s syndrome, or polycystic ovary syndrome; 8) anyone with mental health problems such as severe depression; 9) anyone who was unable to cooperate or to complete the study because of any other reason; and 10) anyone who had participated in another clinical study in the previous 3 months.

### Study plan

#### Run-in period

After successful enrollment, the participants will have a 1-week (±2 days) run-in period, during which they will be required to record their diet and exercise using the smartphone application MyFitnessPal on a daily basis. Their compliance will be assessed against this record and participants with poor compliance in the run-in period will not be allowed to continue to participate.

#### Randomized grouping

After the run-in period, the participants will be randomly assigned to the high-protein diet and resistance training combination group (*n* = 30), the high-protein diet group (*n* = 30) or the conventional low-fat diet control group (*n* = 30) in a 1:1:1 ratio. The high-protein diet group will consume 900–1700 kcal per day, with 25–35% carbohydrate, 30–40% protein and 30–40% fat, and their daily protein intake per kilogram of fat-free mass will be 1.5–2.0 g. The conventional low-fat diet group will also consume 900–1700 kcal per day, but the proportion of carbohydrate, protein and fat will be 50–60%, 15–20% and 25–30%, respectively, and their daily protein intake per kilogram of fat-free mass will be 0.8–1.2 g. The details of the resistance training are shown in Additional file 1. All participants will take part in aerobic exercise as a background intervention because of its necessity in weight loss. Similarly, because of the importance of dietary intervention in weight loss, the study does not include a group who just perform resistance training without any dietary intervention.

#### Intervention period

The participants’ baseline data will be collected, including body weight, body fat percentage and fat-free mass (using the Tanita TBF-300A Body Composition Analyzer, Tanita Europe, UK), waist circumference, hip circumference, systolic and diastolic blood pressure (Omron sphygmomanometer, Omron Healthcare Limited, Milton Keynes, UK), and a blood sample for the determination of blood glucose, blood lipids, renal function and insulin level. These will be collected in the morning after an 8-h fast.

During the 8-week intervention, the participants will receive dietary guidance, which varies by group, and prepare their own food following advice from the dietician. Participants in the resistance training group have to take part in resistance training at least three times a week. During the 8 weeks, the participants will receive face-to-face visits in weeks 2, 4 and 6, and their body weight, waist circumference, hip circumference, and systolic and diastolic blood pressure will be measured during each visit. The participant will receive telephone calls during weeks 1, 3, 5 and 7. During both visits and telephone calls, dietary and exercise guidance will be provided and recorded, and the MyFitnessPal diet inputs will be checked and recorded in written case report forms. The participants will also receive a face-to-face visit at the end of the 8-week intervention, and their body weight, body fat percentage and fat-free weight, waist circumference, hip circumference, and systolic and diastolic blood pressure will be measured, and blood samples taken for the determination of blood glucose, blood lipids, renal function and insulin levels. The participants’ self-efficacy will be investigated using the General Self-Efficacy Scale [24], and the diet management knowledge will be evaluated using a self-designed questionnaire.

During the intervention period the participants will not be allowed to add anything to their agreed intake (such as snacks and drinks) or to change their diet. If the participants wish to change their main meal for a particular reason (such as a social event), the investigator will be consulted by telephone to decide on the remedial measures required (such as reduction of calorie intake the next day). Any changes will be recorded using the MyFitnessPal record.

#### Follow-up period

During the follow-up period, participants will no longer be required to continue with their diet and training plan, although this will be strongly recommended by the investigators until the achievement of individualized weight loss goals. The participants will receive a telephone follow-up every 4 weeks when their body weight and waist circumference will be measured by the participants themselves and recorded by the investigators. Face-to-face visits will be also carried out at the end of the 24-week observation period. Body weight, body fat percentage and fat-free mass, waist circumference, hip circumference, and systolic and diastolic blood pressure will be measured, diet will be investigated, and blood samples will be taken for the determination of blood glucose, blood lipids, renal function and insulin levels.

During the baseline period and throughout the trial the participants will be asked to maintain a flexible diet, pay attention to the type, quantity and weight of their food and liquid intake, and use the smartphone application MyFitnessPal to record their daily diet in detail. This is a reliable way to track diet [25–27]. Throughout the study, the investigators will use WeChat, telephone or MyFitnessPal to monitor and guide the participants on diet and training, ensure strict compliance with plans, and provide extra guidance to anyone with poor compliance. Participants with poor compliance will be allowed to continue to participate but will be warned about the importance of compliance. Diet will be evaluated using the professional diet analysis software Nutritics (Nutritics Ltd., Dublin, Ireland). The participants’ daily calorie intake will be assessed using the Katch-McArdle formula, which is based on the resting daily energy expenditure (RDEE; 370 + (21.6 × lean body weight in kg)) and adjusted for exercise needs and nonexercise adaptive heat generation [28]. The calorie intake assessment will ensure that participants have a negative calorie balance of 500–700 kcal per day during the study period, so the daily recommended energy intake (kcal) will be 1.2 (or 1.3) × RDEE − 500 (or 700). Daily protein intake in the high-protein diet group = fat-free mass × 1.5–2.0 (g), and the daily protein intake in the conventional protein diet group = fat-free mass × 0.8–1.2 (g).

#### Outcomes

The primary outcome is body weight change at week 8 and week 24 compared with the baseline level.

Secondary outcomes include:

1. Change in body fat percentage at weeks 8 and 24 compared with the baseline level
2. Change in waist circumference at weeks 8 and 24 compared with the baseline level
3. The proportion of participants who have successfully lost weight and been nonobese (defined as BMI <24 kg/m^2^) at weeks 8 and 24
4. Diet management ability and weight loss perseverance at weeks 8 and 24
5. Change in self-efficacy at weeks 8 and 24 compared with the baseline level
6. Compliance with the original diet plan at weeks 8 and 24
7. Change in fasting blood glucose at weeks 8 and 24 compared with the baseline level
8. Change in fasting insulin level at weeks 8 and 24 compared with the baseline level
9. Change in blood pressure at weeks 8 and 24 compared with the baseline levels
10. Incidence of adverse events

### Efficacy assessments

Body weight is the primary outcome of this study and is selected as the main variable. Some important secondary variables with clinical significance are also selected for evaluation. Examinations conducted at each visit are shown in Table 1.

**Table 1:** Study plan detailing the procedures

Study period	Screening	Intervention period								
Visit (V)	V1	V2	V3	V4	V5	V6	V7	V8	V9	V10
Study week	−1 (±2 days)	Randomization	1 (±2 days)	2 (±2 days)	3 (±2 days)	4 (±2 days)	5 (±2 days)	6 (±2 days)	7 (±2 days)	8 (±2 days)
Telephone visit			√		√		√		√	
Screening/demography/baseline										
Written informed consent	√									
Inclusion/exclusion criteria	√	√								
Demographics	√									
Physical examination, height, and weight	√									
Medical/ current conditions	√									
History of diabetes and complications	√									
Intervention										
Instruction of diet and exercises		√	√	√	√	√	√	√	√	
Vital signs (blood pressure, heart rate)	√	√								√
Body weight	√	√	√	√	√	√	√	√	√	√
Waist circumference, hip circumference	√	√	√	√	√	√	√	√	√	√
Body fat percentage, lean body mass		√								√
Hematology panel		√								√
Urinalysis		√								√
Liver function		√								√
Creatinine, uric acid		√								√
Lipids		√								√
Glucose, insulin		√								√
Adverse events		√	√	√	√	√	√	√	√	√
Diet management knowledge										√
Self-efficacy scale		√								√

#### Randomization and allocation concealment mechanism

The stratified block randomization method will be used. Participants will be stratified by research center, and the appropriate segment lengths will be selected. Based on the number of seeds, a random coding table of 90 participants will be generated using the statistical software SAS8.2 PROC PLAN by a third party (ZT and LS). The randomized sequential numbers will be segmented, retained in the research centers, and managed by the third party (ZT and LS) who will not be involved in data collection. The investigators (SX, JZ, RC and WX) will enroll participants. When needed, the investigator (SX) will ask for numbers from the third party by telephone, using the order in which the participants will be treated, and assign participants to the intervention scheme with that serial number provided.

#### Blinding methods

This is an open-label study with only laboratory personnel and data analysts (the third party, ZT and LS) being blinded. Unblinding will not occur.

#### Statistical analysis

Epidata 3.1 (The EpiData Association, Odense, Denmark) will be used to manage data, and double data entry will be used to promote data quality. The primary and secondary outcomes will be analyzed based on the full analysis set. All outcomes will also be analyzed based on the per-protocol set. The security end point will be analyzed based on the safety analysis set. Supportive sensitivity analysis will be carried out based on the per-protocol and full analysis set using the method of last observation carried forward. Descriptive analysis will be conducted for continuous variables such as body weight, body fat percentage, blood glucose, blood lipids and blood pressure. Indexes such as the percentage of participants achieving effective weight loss will be summarized using frequencies and percentages. The adverse events and their incidence will be summarized. The mid-term analysis will be carried out at the end of the intervention, before the follow-up study.

#### Determination of sample size

This study is an exploratory pilot study. In line with previous studies [18, 23], 30 participants in each group were initially scheduled to evaluate the efficacy of each group, whether more participants should be included, or whether the study should be stopped.

## Discussion

Generally speaking, an energy imbalance causes obesity or overweight. Only a negative energy balance can result in weight loss. To lose weight, therefore, people require an energy intake that is always lower than their energy consumption [29]. Diet and exercise are ways to control energy intake and consumption, and the basic strategy to lose weight is diet control, exercise, or a combination of the two. In addition to calorie restrictions, a low-fat diet is also considered to be important in weight loss because different nutritional ratios have different effects on satiety, fat-burning and maintaining fat-free mass (muscle) with metabolic activity. As well as aerobic exercise, resistance training has been found to reduce fat, which plays an important role in maintaining fat-free mass and high metabolic activity.

There have been some international studies on short-term weight loss and long-term weight maintenance associated with a high-protein diet [18]. The meta-analysis by Clifton et al. included 32 studies and compared high-protein and low-carbohydrate diet with a normal-protein weight-loss diet. The results showed that most intensive interventions based on a short-term high-protein diet were accompanied by better weight loss and fat content reduction. A protein content difference of more than 5% led to three times more weight being lost (0.9 versus 0.3 kg) in 12 months, and the high-protein diet had better effects on fasting triglycerides and insulin. Unfortunately, there is very limited high-quality research on the effect of a high-protein diet on weight loss in China. Chen et al. [19] observed the effect of a high-protein diet on weight loss in an obese or overweight Chinese population with hyperlipidemia. The average weight loss and BMI decrease percentage in the high-protein group was higher than in the normal-protein group at week 12, but the difference was not significant. There was a significantly larger decrease in the waist to hip ratio in the high-protein group than in the normal-protein group. The triglyceride level decreased in both groups and showed no significant difference between the two groups. These results suggested that a high-protein diet for overweight or obese Chinese people with hyperlipidemia could lead to a more significant reduction in waistline size. However, the study only observed the effect on short-term weight loss and did not assess the effect of a high-protein diet on medium- and long-term weight maintenance [19].

More importantly, both internationally and domestically, few studies have focused on the role of resistance training in medium- and long-term weight maintenance, and the results are inconsistent [20–23]. Some studies have reported that resistance training contributed to the maintenance of weight loss [22], some that it has an effect on the maintenance of fat reduction [30], and one that there was no significant difference in the maintenance of weight loss and fat loss with resistance training [23]. The heterogeneity of study populations and study designs may partly explain the differences between the results. For example, one study included men aged 35–50 years old, with a 6-month intervention period, while another included premenopausal women aged 21–46 years with a weight loss of 12 kg, and a follow-up period of 1 year.

Regaining weight after effective short-term weight loss is often a problem, especially in participants of studies providing centralized meals in the early stage. These studies have found that, after the centralized diet was stopped, a significant proportion of the participants were unable to adhere to the original diet. Studies have shown that the establishment of self-management of dietary intake seems to be a key factor, and the formation of good dietary management habits in the initial weight loss stage will help participants to avoid regaining weight. The weight regained was higher for participants receiving a lower initial calorie intake [31]. One study showed that weight lost and regained depended more on high-protein than on low-carbohydrate levels in the diet and had no significant association with dietary fat [32].

Few studies have explored whether a combination of resistance training and diet will achieve better results than just one method [22, 30, 33]. For obese people, sports, especially resistance training, are difficult because they require extra money and time, and may require participants to attend particular locations [34]. Studies have found that guided exercise could improve weight maintenance among obese people who would otherwise take no exercise [30].

To achieve successful long-term weight loss, the intervention must effectively improve the individual’s self-efficacy. Improvements in self-efficacy are strongly affected by the management of dietary intake and the enthusiasm for exercise; participants who regain weight are more likely to give up and lose motivation [17]. One of the core requirements of resistance training is to persist, so it may have a better effect on perseverance than traditional aerobic exercises such as fast walking or jogging. In this study, we assumed that resistance training can increase the proportion of subjects who adhere to the original diet (or decrease the degree of noncompliance), and therefore ultimately improve the long-term weight loss effect to avoid regaining weight.

Overweight and obesity are worldwide problems, and the exploration of scientific weight management and weight loss schemes has been an important area of research and has attracted a lot of investment. However, appropriate weight loss and weight maintenance strategies in specific populations (such as different ethnic groups) and social environments are unclear. We hope that the PROMOTE study will provide a basis for obesity management in China and promote the development of exercise- and diet-related studies. These are important to improve nutritional status, promote healthy development, prevent obesity-related diseases, and improve the health and quality of life of the population.

## Trial status

The trial is currently recruiting participants. The recruitment began in June 2019 and is anticipated to end in October 2020. The trial is registered in the Chinese Clinical Trial Registry (https://www.chictr.org.cn/) with registration number ChiCTR1900023841, registered on 14 June 2019.

## Supplementary information

**Additional file 1.** The details of the resistance training.

## Data Availability

The datasets used and/or analyzed during the current study are available from the corresponding author on reasonable request.

## Abbreviations

BMI: Body mass index
PROMOTE: High-protein and resistance-training combination in overweight and obesity
RDEE: Resting daily energy expenditure
