# Supplementary material for: The PROMOTE study (High-protein and resistance-training combination in overweight and obesity) for short-term weight loss and long-term weight maintenance for Chinese people: a protocol for a pilot randomized controlled trial
Source: Trials. 2020 Jan 8;21:47. doi: 10.1186/s13063-019-3954-7 (PMC6950796; doi:10.1186/s13063-019-3954-7)
Supplement: Supplementary file 1 — Additional file 1. The details of the resistance training. [file 13063_2019_3954_MOESM1_ESM.docx]

**Additional file 1**

**Exercise prescription and content:** aerobic exercise: 10 min warm up and stretch to warm up the body and to avoid injuries. Aerobic training included treadmill jogging and uphill brisk walking, elliptical cross trainer and spinning. The training intensity was based on heart rate: the maximum heart rate (BPMmax) was calculated as 220 − age, and the aerobic heart rate range was 60%–80% of BMPmax. The exercise intensity was adjusted gradually by heart rate, and the total exercise time was 35 minutes.

**Resistance training:** once participants had fully warmed up, the resistance training combined major and minor muscle groups, divided into three categories: chest muscle group and triceps brachii muscle; back muscle group and biceps brachii muscle; and leg muscle and shoulder muscle group. Each major muscle group was exercised once a week, requiring at least three exercise sessions per week. Each exercise session lasted about 35 minutes. The principles of the resistance training were targeting, load sequence, incremental load, reasonable resistance combined with aerobic exercise, comprehensive program and safety. The first week was the adaptation period (using small load training). The one-repetition maximum (1RM) test was carried out first, and each muscle group was exercised and 65% 1RM for 8–12 times per group over 2–4 repetitions (excluding the warm-up). The weight was gradually increased over the next few weeks until an 80% 1RM load was reached in the fourth week. From week 5 to week 8, participants did 80% 1RM for 8–12 times/group over 2–4 repetitions (excluding the warm-up). The resistance training plan was based on the recommendation of the American College of Sports Medicine [5], and was guided by a personal trainer with a coaching certificate. Specific items are shown in Table 1.

**1RM test:** 1RM is the maximum load that can be successfully completed once. During the test, the initial load of different muscle groups should usually be lower than the 1RM load, and a higher load can be tested after successfully completing the initial load and resting for 2–3 minutes, until a 1RM load is reached. In general, depending on the muscle group measured, it is necessary to increase the weight by more than 1.5 kg at a time to ensure the accuracy and safety of the test. Methods: warm up on treadmill or elliptical cross trainer for 5 minutes, and then perform the one-repetition maximum (1RM) test; the following actions can be taken: squat, chest press and bent over row. For each training action, the participant first had to warm up (40% 1RM, 10 times), then increase to 60% 1RM (5 times), 80% 1RM (1–2 times), and finally 1RM (once). 5 minutes rest was allowed between efforts. Resistance training was required at least three times a week.

**Table S1. Training items for different body parts**

| Training items | |
| --- | --- |
| Chest | Flat bench press; incline bench press; cable iron cross, seated cable fly, dumbbell fly (choose one item); decline bench press. |
| Shoulder | (Choose three items) Machine shoulder presses, incline dumbbell press, barbell military press (choose one item); dumbbell lateral raise; incline bench reverse flyes, reverse machine flyes, cable reverse fly, cable V bar pull down (choose one item) |
| Back | (Choose three items) Wide-grip lat pull-down, narrow-grip lat pull-down, pull up, seated cable rows, bent over two-dumbbell row |
| Legs | (Choose three items) squat, leg press, lunge, leg curl, leg flexion, calf raise, stiff-legged deadlift |
| Triceps brachii | (Choose two items) Cable rope triceps extension, skull crusher, French press |
| Biceps brachii | (Choose three items) Cable biceps curl, dumbbell bicep curl, preacher dumbbell curl, machine preacher curl, seated preacher curl, balance push-up, standing biceps curl, swiss-ball seated curl, Zottman curl |
